# Post‐mortem CT detection of fatal air embolism after aerosolized fibrin glue for bladder bleeding

**DOI:** 10.1111/1556-4029.70278

**Published:** 2026-02-05

**Authors:** Beatrice Benedetti, Nazario Foschi, Caterina Pesaresi, Tommaso Tartaglione, Matteo Mancino, Alberto Chighine, Fabio De‐Giorgio

**Affiliations:** ^1^ Fondazione Policlinico Universitario A. Gemelli IRCCS Rome Italy; ^2^ Department of Healthcare Surveillance and Bioethics, Section of Legal Medicine Università Cattolica del Sacro Cuore Rome Italy; ^3^ Department of Urology Fondazione Policlinico Universitario Agostino Gemelli IRCCS Rome Italy; ^4^ Department of Radiodiagnostic Fondazione Policlinico Universitario A. Gemelli IRCCS Rome Italy; ^5^ Department of Medical Sciences and Public Health, Section of Legal Medicine University of Cagliari Cagliari Italy

**Keywords:** air embolism, fibrin glue, forensic autopsy, post‐mortem computed tomography, radiation‐induced hemorrhagic cystitis, transurethral resection of the bladder

## Abstract

Radiation‐induced hemorrhagic cystitis (RHC) is a severe complication of pelvic radiotherapy, often used to treat various pelvic malignancies. Despite multiple therapeutic options, including conservative and invasive interventions, the optimal management remains uncertain. We report the case of a 76‐year‐old male with pulmonary emphysema and a history of prostate cancer treated with radiotherapy, who developed refractory RHC. During a hemostatic transurethral resection of a bladder tumor, autologous fibrin glue was applied via aerosol. Shortly after, the patient experienced sudden cardiorespiratory arrest and died. Post‐mortem computed tomography (PMCT) revealed extensive intravascular gas in the heart and cerebral vessels, confirming fatal air embolism. No gas was identified in the pulmonary arteries, and autopsy findings excluded structural cardiac anomalies such as a patent foramen ovale. These results support the hypothesis of a right‐to‐left functional pulmonary shunt, a mechanism in which venous gas bypasses the pulmonary filter through intrapulmonary arteriovenous anastomoses. Pulmonary emphysema, present in this case, may have contributed by impairing alveolar‐capillary integrity and reducing vascular filtration capacity. Additionally, bladder adhesions observed at autopsy likely reduced bladder compliance, facilitating air entry during glue application. This is, to our knowledge, the first documented case of fatal air embolism following aerosolized fibrin glue use for RHC, confirmed by both PMCT and autopsy. The case highlights the need for caution when using aerosolized hemostatic agents in patients with predisposing factors such as bladder adhesions and obstructive pulmonary diseases. Furthermore, it demonstrates the essential role of PMCT in identifying embolic complications and determining the cause of death in forensic settings.

**Highlights**

- RHC is a severe complication of pelvic radiotherapy that lacks a unified treatment strategy.
- Aerosolized fibrin glue may lead to fatal air embolism during bladder hemostasis.
- PMCT demonstrated cerebral and cardiac air embolism with no signs of putrefaction.
- Pre‐existing conditions such as bladder adhesions and eroded vessels increase embolic risk.

## 1 INTRODUCTION

Actinic cystitis is one of the most common complications of pelvic radiotherapy. The radiation used for therapeutic purposes causes both acute and chronic secondary damage. The etiology of this phenomenon can be attributed to the direct damage inflicted by radiation energy on outer‐layer urothelial cells, as well as to indirect damage stemming from the generation of free radicals following radiation exposure. Patients may experience actinic cystitis both shortly after radiotherapy and a long time after [1].

Actinic cystitis can be complicated by hemorrhage, resulting in radiation‐induced hemorrhagic cystitis (RHC). This results from urothelial and microvascular damage caused by the physical effects of radiation. According to recent studies, this process triggers neoangiogenesis, resulting in the formation of new fragile vessels and subsequent susceptibility to bleeding [2].

There is no consensus regarding the clinical management of RHC. The guidelines recommend cystoscopy to exclude malignancies; however, subsequent therapeutic strategies lack a clear direction. Common interventions include the intravesical administration of hyaluronic acid, oral administration of pentosan polysulfate, nonsteroidal anti‐inflammatory drugs, amitriptyline, and other oral medications, as well as hyperbaric oxygen therapy [3]. In more severe cases or when bleeding becomes the primary concern, invasive procedures, such as endoscopic cauterization, endovascular internal iliac artery embolization, or salvage cystectomy with urinary diversion, are the last resort [4]. Among these interventions, the use of fibrin glue as a topical hemostatic treatment to alleviate and resolve bladder bleeding has been reported [5, 6].

This therapy is typically considered a second‐line option reserved for cases in which conservative treatments have failed [7]. Currently, there is insufficient data on its efficacy, safety, and long‐term follow‐up; as a result, it has not been included in the latest scientific recommendations [8].

We present the case of a 76‐year‐old man who died from an air embolism resulting from autologous fibrin glue treatment for refractory RHC. To our knowledge, this is the first case in which a fibrin glue‐induced air embolism was investigated using both autopsy and post‐mortem computed tomography (PMCT).

## 2 CASE REPORT

A 76‐year‐old male patient with a remote medical history of prostate cancer and pulmonary emphysema was treated with radical prostatectomy and radiotherapy approximately 20 years prior. Owing to massive hematuria and dysuria, he underwent cystoclysis and subsequent cystoscopy which revealed actinic cystitis. Hemostatic transurethral resection of bladder tumor surgery was planned for both diagnostic and therapeutic purposes, including the simultaneous application of autologous fibrin glue for hemostasis delivered through aerosol application (Vivostat® Fibrin Sealant, Vivolution A/S, Birkerod, Denmark). During surgery, CO_2_ was insufflated into the bladder at a pressure of 12 mmHg. Following the application of 6.1 mL of fibrin glue, the patient suddenly developed bradycardia, with his heart rate dropping to 50 beats per minute, followed by loss of consciousness and asystole. Despite orotracheal intubation and resuscitation, the resuscitation was not successful. Thoracic and abdominal ultrasonography performed during resuscitation excluded pneumothorax and hemoperitoneum.

A complete autopsy was performed 7 days after death, immediately following a preliminary whole‐body computed tomography (CT) scan. The body had been refrigerated at a temperature of +4°C during this period.

A PMCT examination was conducted using a 16‐slice Somatom Scope CT scanner. Axial volumetric acquisitions were obtained with multiplanar reconstructions. The images were interpreted by a radiologist experienced in forensic pathology.

**FIGURE 1:**
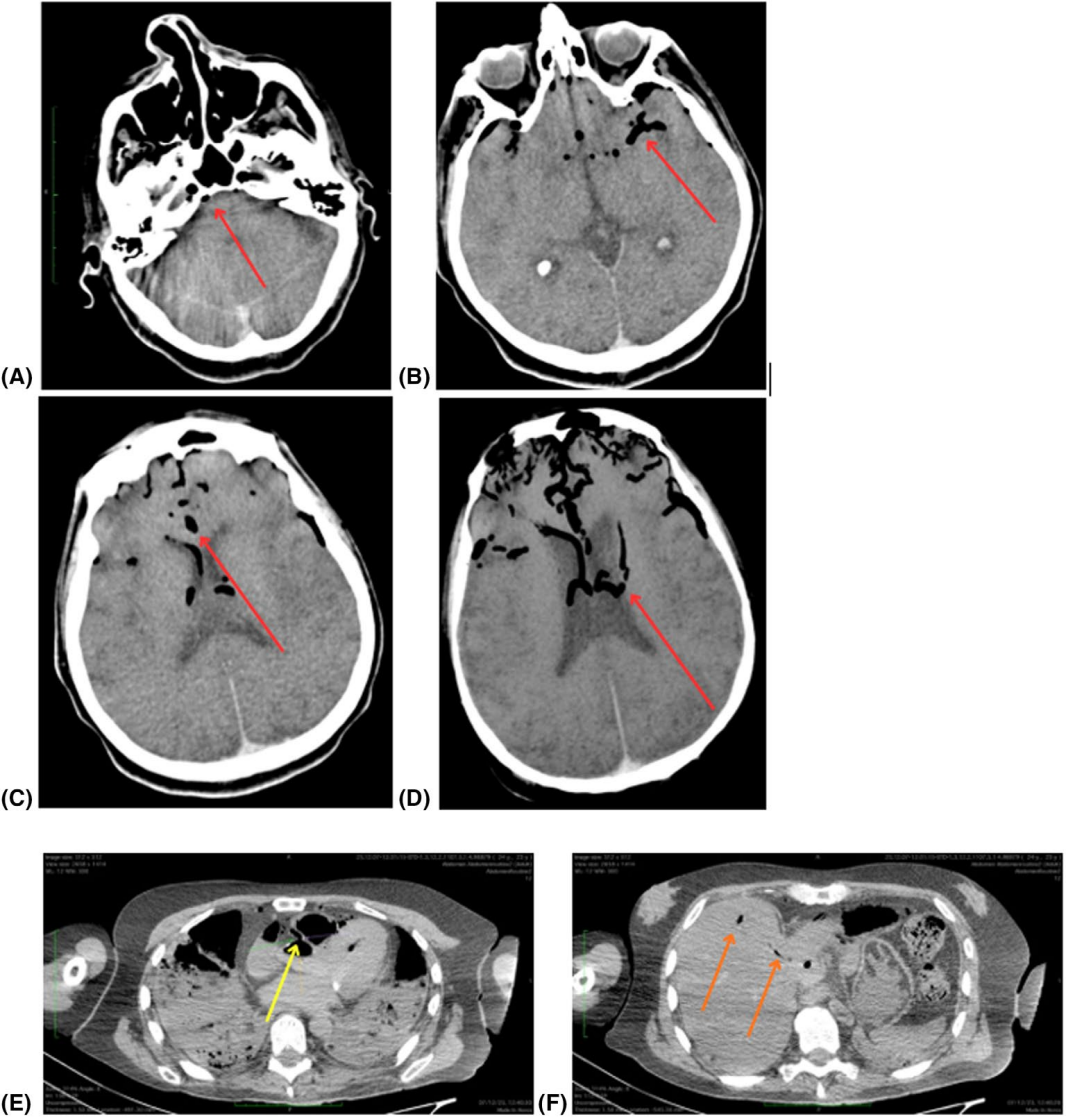
(A–D) Axial brain CT images showing gas within multiple intracranial vessels (red arrows): Basilar artery (A), middle cerebral artery (B), anterior cerebral artery (C), and internal cerebral veins (D). (E) Axial CT image at the level of the ascending aorta and coronary common trunk (yellow arrow). (F) Axial CT image of the upper abdomen showing minimal gas in the left hepatic lobe (orange arrows).

PMCT revealed an unusual gas distribution pattern, characterized by a significant accumulation of gas within the brain and heart. A large amount of intravascular air with fluid–air levels was observed in the right atrium and ventricle, left ventricle, coronary trunk, ascending aorta, and aortic arch, as well as in both common carotid arteries and the right brachiocephalic trunk. Minimal gaseous content was detected in the left hepatic lobe, with no air in the right hepatic lobe and no evidence of pneumoperitoneum (Figure 1). In addition, abundant air was present within the ureters and renal pelvis. Diffuse calcific parietal deposits were noted along the aortic and iliac walls, as well as along the course of the coronary arteries.

On external examination, no signs of ongoing putrefaction were observed, consistent with the continuous refrigeration of the body.

During the autopsy, the brain was examined through serial coronal sections from the frontal to the occipital poles and showed no pathological lesions. The circle of Willis was also intact, with no evidence of vascular abnormalities. Additional relevant findings included pulmonary emphysema with a large apical bulla in the left upper lobe. Examination of the coronary arteries revealed diffuse non‐occlusive calcified plaques in the left anterior descending and circumflex arteries, as well as in the proximal segment of the posterior descending artery. A water immersion test was performed to evaluate the presence of pulmonary arterial air emboli, resulting in the release of a few microbubbles.

The urinary bladder showed dense adhesions without wall perforation, bilateral hydronephrosis, and an enlarged right caval lymph node. Findings consistent with RHC were observed in the bladder wall.

No significant pathological changes were observed in the remaining organs.

Histological examination revealed cerebral edema and diffuse waviness of myocardial fibers consistent with terminal ischemia, with no evidence of autolytic or putrefactive changes. The lung tissue confirmed severe pulmonary emphysema, while the bladder showed diffuse erosive chronic cystitis with mural hemorrhagic changes. The right caval lymph node contained metastatic deposits from a differentiated adenocarcinoma.

## 3 DISCUSSION

We report a rare case of death in a patient with RHC following radiotherapy for prostate cancer, resulting from cardiorespiratory arrest secondary to cardiac and cerebral ischemia induced by air embolism as a complication of bladder fibrin glue application. The uniqueness of this case lies not only in its rarity but also in the reconstruction of the cause of death, which was strongly supported by the findings obtained through PMCT.

The use of PMCT is well‐established in forensic investigations due to its numerous advantages, particularly in cases involving blunt force trauma, gunshot wounds, personal identification, and estimation of the post‐mortem interval [9, 10, 11].

In our case, PMCT allowed for the identification of a gas distribution pattern highly suggestive of embolism, which would have been challenging to diagnose through traditional autopsy alone.

More specifically, the PMCT scan revealed two anatomical regions that were particularly affected by embolic phenomena: the heart and the brain. In the evaluation of gas distribution patterns, it is crucial to differentiate between post‐mortem gas formation due to putrefaction and ante‐mortem air embolism. It is well established that the chemical processes involved in decomposition generate volatile compounds, with their generation varying according to the types of microorganisms active during the different stages of putrefaction, as well as the environmental conditions [12]. In the differential diagnosis of these two conditions, putrefactive gas is typically found in greater amounts within deep organs, such as the liver, and in the vessels of the gastrointestinal tract [13, 14]. In our case, the examination of hepatic imaging showed minimal gaseous content in the left hepatic lobe. This confirmed the absence of significant putrefactive alterations, which is a crucial aspect that must always be carefully evaluated to exclude artifacts [15].

An additional confounding factor in the interpretation of PMCT findings is the distribution of gas associated with cardiopulmonary resuscitation (CPR), particularly when performed for a prolonged period [16, 17]. Previous studies have demonstrated that, in such instances, gas accumulation is predominantly limited to the venous system [18]. In the present case, a significant contribution of resuscitation‐related gas was excluded based on both the anatomical distribution revealed by PMCT—which extended beyond the venous system—and the clinical context. Specifically, CPR was performed in accordance with standardized protocols and for a conventional duration. Furthermore, ultrasonographic examinations carried out during resuscitation ruled out pneumothorax and hemoperitoneum, thereby supporting the diagnosis of genuine air embolism.

Air embolism is a rare but potentially fatal event that can occur when air enters the venous or arterial bloodstream; it can result from a variety of procedures and clinical scenarios [19]. One cause of air embolism is the presence of a negative pressure gradient in blood vessels, which is commonly associated with central venous catheterization. Additionally, positive‐pressure gas insufflation can create a pressure gradient that poses a high risk of gas embolism [20], as observed in the present case.

Another contributing factor is the presence of eroded blood vessels, in which air entry is facilitated by the discontinuity of the vessel wall [21]. In most cases, air embolisms manifest clinically; however, some instances of venous air embolism remain asymptomatic [22]. The main clinical manifestations include hypotension, dyspnea, and heart rhythm disturbances, such as tachycardia, bradycardia, and asystole [23].

The findings from both autopsy and virtual autopsy in our case indicate a potential additional source of embolism aside from the CO_2_ necessary for laparoscopy: the entry of air produced by the positive pressure insufflation of fibrin glue using a specialized applicator. When these considerations are applied to the specific circumstances of our case, we believe that this mechanism represents the most plausible cause of the air embolism. This interpretation is supported by the fact that the bladder insufflation pressure of 12 mmHg falls well within the normal operational range for such procedures [6, 24] and, on its own, would be unlikely to account for such a severe complication. Experimental animal studies have in fact identified a threshold value of approximately 30 mmHg, above which the risk of air embolism during abdominal laparoscopic procedures significantly increases [25]. We hypothesize that a significant risk factor in our case was the erosion of the bladder mucosa and blood vessels, characteristic of RHC, which increased the risk of air passage into the systemic circulation. This hypothesis is supported by PMCT findings showing an abnormal distribution of air bilaterally and symmetrically in the carotid arteries, basilar artery, middle and anterior cerebral arteries, internal cerebral veins, and bilateral temporal and fronto‐basal prepolar cerebrospinal fluid space.

The absence of gas embolism within the pulmonary vasculature on PMCT led to the consideration of two potential mechanisms: a right‐to‐left intracardiac shunt or a functional pulmonary bypass. The first hypothesis was excluded based on autopsy findings, which revealed no anatomical anomalies of the heart—such as a patent foramen ovale or other structural defects—that could account for the paradoxical embolism.

The second hypothesis, a right‐to‐left functional shunt, appears more plausible. Literature reports have described the potential for venous gas to bypass the pulmonary circulation through physiological intrapulmonary arteriovenous anastomoses (IPAVAs), whose flow can vary depending on physiological stimuli such as physical exertion, hypoxia, or hyperoxia [26]. An additional factor known to influence IPAVA flow is the presence of obstructive pulmonary diseases. Indeed, it has been demonstrated that individuals with chronic obstructive pulmonary disease (COPD) exhibit increased baseline IPAVA perfusion compared to healthy individuals, even at rest [27].

In the present case, this mechanism may have been facilitated by the patient's underlying pulmonary emphysema, a major component of COPD, with which it shares key pathophysiological features—such as destruction of alveolar walls, impaired alveolar‐capillary integrity, and reduced efficiency of the pulmonary vascular filtration system [28]. These alterations may have contributed to the formation of a functional pulmonary shunt, ultimately allowing gas emboli to reach the systemic circulation and cause widespread embolic injury.

The resulting damage to the brain and heart likely led to rapid death because both circulatory systems are highly susceptible to ischemic injury [29].

Another element worth highlighting is the presence of bladder adhesions, as evidenced during the autopsy, which were likely a consequence of pelvic radiotherapy toxicity. We believe that these adhesions may have led to reduced bladder compliance upon the application of exogenous pressure because of the decreased elasticity caused by fibrotic tissue [30]. This anatomical alteration could be a significant risk factor for embolic complications, particularly considering that they are known complications of procedures involving the application of fibrin glue for hemostasis. Specific risk factors include excessively high application pressures and short distances from the applicator when spraying the glue. Unfortunately, the device used in our case does not allow for adjustment of the application pressure of the fibrin glue, which we consider a technical limitation that warrants future refinement.

In comparison with our case, Ebner et al. described the case of a 45‐year‐old woman who underwent partial liver resection for metastasis where fibrin glue treatment was used to treat hemorrhage [31]. Immediately after the procedure, the patient developed severe hypotension and bradycardia, requiring cardiopulmonary resuscitation. Subsequently, her cardiac function improved, and she was discharged approximately 2 weeks later without any complications. The cause of the complication was demonstrated through echocardiography, which revealed the presence of air in the ventricles and a floating thrombus in the right ventricle.

To the best of our knowledge, this is the first documented instance of death due to air embolism as a complication of fibrin glue application in a patient with RHC, as evidenced by post‐mortem imaging.

We believe that our findings underscore the importance of investigating the clinical use of aerosolized fibrin glue, particularly its safety parameters, especially in cases where the risk of air embolism is increased, such as in RHC. It is also crucial to note that fibrin glue is currently used in other urological settings, such as BK virus cystitis following renal transplantation [19] or the conservative management of urinary fistulas [32, 33]. Emphasizing how to prevent this potentially fatal complication is important, as it remains unclear whether aerosolization is a critical factor and whether instillation could achieve similar results with reduced risk.

## CONFLICT OF INTEREST STATEMENT

The authors have no conflicts of interest to declare.

## CONSENT FOR PUBLICATION

The individual examined in the current study underwent a forensic autopsy. The collection of data, sampling, and subsequent forensic analyses were conducted with authorization from the Public Prosecutor. Informed consent was obtained through the local Prosecutor's office, which acted in what was believed to be the closest interest of the deceased. The report does not contain personal data. All data are covered by the Italian Law—Data Protection Authority (Official Gazette no. 72 of March 26, 2012)—for scientific research purposes. The study complies with the principles of the Helsinki Declaration and with the requirements of the European Union GDPR regarding consent.

## Data Availability

The data that support the findings of this study are available from the corresponding author upon reasonable request.
